# Sarcoidosis: Yet Another Masquerader The Cardiologist / Electrophysiologist   Has To Fight With

**Published:** 2010-12-26

**Authors:** Syamkumar M Divakara Menon, Diego Chemello, Carlos A Morillo

**Affiliations:** McMaster University and Hamilton health Sciences, Hamilton, ON, Canada

**Keywords:** sarcoidosis, arrhythmias

Sarcoidosis is a multisystem  granulomatous disease  of unknown aetiology characterized by  infiltration of non caseating granulomas in multiple organ systems. Sarcoidosis can have a plethora of cardiac manifestations which can mimic many other cardiovascular conditions ranging from asymptomatic electrocardiographic manifestations to life threatening arrhythmias resulting in sudden cardiac death. Even  more than a century after the first description of the disease by Norwegian dermatologist Caeser Boeck in 1899 [1], the aetiology of the disease remain elusive.

## Pathophysiology  of sarcoidosis

The pathological hallmark of sarcoidosis is the non caseating granuloma. The inciting event  which  leads to the formation of the granuloma is still unknown. various environmental agents have been postulated as  triggers leading to granuloma formation including  propionibacter and mycobacterial infections, tree  pollen, photocopier toner dust, insecticides, vegetable dust and titanium eposure [2-4].

## Cardiac involvement

Depending on the criteria used for diagnosis, cardiac involvement range from 5% to 50% [5,6]. The prognosis of cardiac sarcoidosis is consistently severe despite the variability in  prevalence and  clinical manifestations. It is the second most common cause of sarcoid related mortality after respiratory failure in western countries and probably most common cause of mortality in Japan [7,8].

## Cardiac manifestations

Usual clinical manifestations of cardiac sarcoidosis (CS) include:

1. Conduction abnormalities either symptomatic or asymptomatic. This include atrio ventricular or interventricular conduction abnormalities.
2. Ventricular arrhythmias.
3. Congestive heart failure
4. Sudden cardiac death [5].

Conduction abnormalities are common. Both advanced AV block and ventricular arrhythmias can result in sudden cardiac death. Congestive heart failure has been observed in up to 30% cases [9-11].

Apart from the common presentations, CS can mimic pericarditis, ARVD, and acute coronary syndromes [12,13].

CHF is caused by myocardial infiltration of the sarcoid granulomas and  is typically rapidly progressive over weeks or months. Often there will be associated conduction abnormalities. Valvular regurgitation because of endocardial involvement also can contribute to development of congestive heart failure.

Sudden cardiac death is the most dramatic and feared complication of  CS,  with an estimated incidence of 23 to 66% in different series [7,10,11].

## Diagnostic evaluations

### Electrocardiography

Up to 75% cases of CS will demonstrate ECG abnormalities. Conduction abnormalities are most common, with a prevalence  ranging from 12% to 62%. Bundle branch blocks either complete or incomplete (12-61%) and atrioventricular block (23-60%) are common. Ventricular arrhythmias  are common, and are described in up to 42% cases. Ventricular tachycardias are related to myocardial granulomas and subsequent fibrosis. Supraventricular arrhythmias are less common (0-15%). Atrial arrhythmias are more often due to atrial dilatation secondary to pulmonary involvement rather that atrial myocardial involvement [11]. Less common ECG abnormalities include sinus node dysfunction, repolarisation abnormalities, pseudo infarction Q waves, long QT or U waves.

### 24 hour Holter

Suzuki et al [14] have  reported a sensitivity of 67% and specificity of 80% in diagnosis of CS. More severe conduction disturbances than baseline, detection and characterization of  ventricular arrhythmias are the utilities of 24 hour Holter evaluation.

### Electrophysiological study

The role of programmed electrical stimulation in EP study  in CS is controversial [11].  Studies have yielded conflicting results and were under powered to derive any meaningful conclusions [15,16]. Large prospective studies are needed before recommending the role of diagnostic EP studies in risk stratification of CS.

### Echocardiography

Echocardiographic findings are variable in CS depending on the stage of the disease. It can be normal in early stages of the disease. With disease progression, 14-56% of various echocardiographic manifestations are reported. There is no direct correlation of the ECG abnormalities with echocardiographic abnormalities.

Regional wall motion abnormalities, segmental wall thickness abnormalities either thinning or thickening  especially of the basal interventricular septum, left ventricular aneurysms and in severe cases, dilatation of left ventricle with severe impairment of systolic function of the LV are reported in CS [9,10]. Mitral regurgitations either due to endocarditis or papillary muscle dysfunction is not uncommon. Small pericardial effusion is detected in up to 20% cases [17].

Tissue characterization and myocardial strain analysis using tissue Doppler imaging may also be valuable for diagnosis of CS [18,19]. These new imaging modalities need further evaluation and prospective studies before concluding on their utility in diagnosis in CS.

### Nuclear imaging

Myocardial perfusion scintigraphy   with single photon emission computed tomography (SPECT) and thallium 201(^201^Tl) or  technetium 99m (^99^mTc) have been used for evaluation of CS [1]. The characteristic phenomenon of reverse redistribution differentiates CS from coronary artery disease apart from the absence of  vascular territorial distribution  in the former. Resting perfusion typically show nonspecific segmental areas of decreased uptake  which will disappear or decrease in size during exercise or after administration of vasodilator agents like dipyridamole [20]. The exact mechanism of this phenomenon is not well elucidated, however most likely explanation is the presence of focal reversible micro vascular constriction in coronary arterioles surrounding sarcoid granulomas. Gated SPECT images will provide information about global LV function also.

Amount of reverse redistribution has a correlation with degree of improvement under corticosteroids [21]. Conversely, isolated perfusion defects without clinical cardiac involvement is not associated with prognosis in CS [22,23].

#### ^18^F-Fluorodeoxyglucose Positron Emission Tomography (FDG-PET)

FDG uptake in the myocardium typically show either a focal or focal on diffuse pattern (hot spots). These hot spots are presumably related to the sarcoid granulomas and inflammation.

Small studies from Japan compared FDG-PET with  99m Tc-Sestamibi, Ga and ^201^Tl scintigraphy [24-27]. Overall sensitivity of FDG-PET is superior to other imaging modalites (85-100%). However specificity is slightly lower at 81.5 to 90.3%. There are no systematic prospective studies evaluating the role of FDG-PET in diagnosis  of CS or its superiority over the other nuclear imaging modalities to date.

### Cardiac magnetic resonance imaging (CMR)

Contrast enhanced CMR is now considered to be the diagnostic investigation of choice in suspected CS. It can also be an aid for increasing the yield of endomyocardial biopsy. The CMR imaging appearance depends on the age of the pathological process. In acute granulomatous stage, sarcoid lesions will appear as  areas of high signal intensity on T1 and T2 weighted images [28,29] but can also appear as low intensity signal in T1 weighted images [30]. In the chronic stage of the disease, focal areas of myocardial thinning with contractile dysfunction  may result from scar formation. Myocardial scarring is typically patchy and will not confine to vascular territories. The sensitivity and specificity of CMR  are 100% and 78% respectively in a study conducted in 58 patients by Smedema et al [31]. CMR has probably a better specificity but a  lower sensitivity that FDG-PET [15,32].

CMR can  detect pre clinical cardiac involvement. A prospective study in 81 cases has demonstrated patients with myocardial involvement in CMR had an 11.5 fold higher  rate of deaths than those without myocardial involvement [31]. CMR also has prognostic implications with a positive correlation between the extent of myocardial involvement  as evidenced by late enhancement  with left ventricular dilatation,  ventricular  arrhythmias and impairment of LV function [33-36].

### Endomyocardial biopsy

Because of the patchy nature of the disease, the sensitivity of biopsy is low even in patients with dilated cardiomyopathy. Endomyocardial biopsy will demonstrate noncaseting granulomas in less than 25% cases of CS [37,38].  It is not routinely done as a diagnostic work up for CS.

## Management

Once the diagnosis is made, risk stratification is done based on presence of LV function, ECG abnormalities, degree of granuloma  infiltration and some investigators argue for the role of EP studies to evaluate the risk of sudden cardiac death. Any of the abovementioned high risk markers are sufficient to initiate therapy in view of the progressive nature of the disease and risk of sudden arrhythmic death.

Corticosteroids are the mainstay of drug therapy. There is no survival advantage for patients treated with  high   dose  steroids (> 30 mg ) compared with low dose (<30 mg) [39]. Severity of heart failure, LV end diastolic diameter, and presence of ventricular tachycardia are independent predictors for mortality from SHD. Early  initiation of steroid therapy before development of systolic dysfunction resulted in improved outcome.

Other immunosuppressive therapies (azathioprine, cyclophosphamide, or hydroxy chloroquin) are used for patients who are intolerant to steroids or fail to respond to steroid therapy. Refractory cases may be treated with infliximab, a monoclonal antibody against TNF a. These agents have not been evaluated in any prospective randomized trials involving patients with SHD.

Immunosuppression along with catheter ablation has been found useful in treating ventricular tachycardia storm in patients with CS [44].

### Management of arrhythmias

The utility of anti arrhythmic drugs are not proven in CS  in any randomized trials. AAD may increase the risk of heart block, while amiodarone can cause pulmonary complications in the setting of already compromised respiratory function. In a case series of patients with sarcoidosis associated sustained ventricular tachycardia, anti arrhythmic drugs were associated with a high rate of recurrence of arrhythmia or sudden cardiac death or both. 4 of 7 cases where ICD was implanted received appropriate shocks [40]. ICD implantation should be considered in patients with LV dysfunction.

Utility of programmed ventricular stimulation in identifying high risk patients  is controversial. One study demonstrated an added value of EP study in identifying these patients with high risk of SCD. However there are no large prospective randomized studies available in this scenario.

Surgical therapies such as resection of ventricular aneurysm has been reported [41,42]. Cardiac transplantation is an option for patients with refractory heart failure or arrhythmias, however  recurrence of disease in the allograft has been reported [43].

Results from a multicentric registry demonstrated the efficacy of ablation in controlling the symptoms in CS. The disease process often involves a specific area in the basal right ventricle predisposing to peri tricuspid re entry. Both endocardial and epicardial ablatins were performed in these patients and peri tricuspid re entry VT could be eliminated in 70% of the cases [45].

## Conclusions

CS is a great masquerader  which can mimic many cardiac conditions. Significant ventricular involvement is associated with high risk of arrhythmic death as well as heart failure. Diagnosis  and risk stratification is a challenge and is aided by new diagnostic technologies like CMR and FDG PET studies. Immunosuppressant therapy  is the mainstay of treatment. Many  patients will need cardiac rhythm management devises mostly ICD. Ventricular tachycardia storm and ICD shocks will be a continuing problem for these cases. Electrophysiological studies and ablation of the ventricular tachycardia has been shown promising in people with arrhythmia storm. Role of  prophylactic substrate modificatin to avoid ICD shocks in these subset of patients is unknown and has to be evaluated in prospective clinical trials.
